# Species-Specific Responses of Insectivorous Bats to Weather Conditions in Central Chile

**DOI:** 10.3390/ani14060860

**Published:** 2024-03-11

**Authors:** Annia Rodríguez-San Pedro, Juan Luis Allendes, Tamara Bruna, Audrey A. Grez

**Affiliations:** 1 Centro de Investigación e Innovación para el Cambio Climático (CiiCC), Facultad de Ciencias, Universidad Santo Tomás, Avenida Ejército 146, Santiago 8320000, Chile; 2 Departamento de Ciencias Biológicas Animales, Facultad Ciencias Veterinarias y Pecuarias, Universidad de Chile, La Pintana, Santiago 8820000, Chile; agrez@uchile.cl; 3 Bioecos E.I.R.L, Las Condes, Santiago 7591313, Chile; jrallend@gmail.com; 4 Programa para la Conservación de los Murciélagos de Chile (PCMCh), Santiago 8370003, Chile; 5 Centro de Investigación Austral Biotech, Facultad de Ciencias, Universidad Santo Tomás, Avenida Ejército 146, Santiago 8320000, Chile; tbruna@santotomas.cl

**Keywords:** bat activity, Chiroptera, temperature, relative humidity

## Abstract

**Simple Summary:**

As climate change intensifies, understanding the impact of environmental factors on bat activity becomes essential for their conservation. This study, centered in central Chile, utilizes data from automatic bat detectors and climatological stations to explore the effects of air temperature and relative humidity on the timing of activity and abundance of five bat species. Temperature exerts influence over the timing of activity and relative abundance, revealing species-specific responses. Relative humidity emerges as a significant factor affecting abundance, with drier nights exhibiting higher bat pass rates. These findings suggest that global warming has the potential to reshape bat behaviors, impacting foraging patterns and activity levels. Further studies are essential to provide deeper insights that can guide effective conservation efforts in the context of climate change.

**Abstract:**

Insectivorous bats play a crucial role in agroecosystems by providing invaluable pest control services. With the escalating impacts of climate change, a comprehensive understanding of the environmental factors influencing bat activity becomes imperative for their conservation in agricultural landscapes. This study investigates the influence of weather conditions, specifically air temperature and relative humidity, on the timing activity and the relative abundance of five insectivorous bat species in central Chile. Data from automatic bat detectors and climatological stations are utilized for analysis. Our results unveil species-specific behaviors, with *Tadarida brasiliensis* exhibiting early emergence and extended activity periods compared to other bat species. *Histiotus montanus* and *Lasiurus villosissimus* display delayed onsets on more humid evenings, whereas *Lasiurus varius* and *T. brasiliensis* initiate activity earlier on colder nights compared to warmer ones. Relative humidity emerges as a key factor influencing relative abundance for all species, with more minutes with bat passes detected on drier nights. These findings suggest that global warming may influence observed bat behaviors, potentially altering foraging patterns and activity levels of these bat species. Moreover, as climate change continues, understanding the long-term impact on bat populations and their adaptive strategies is crucial for effective conservation measures. Further studies exploring these dynamics can provide valuable insights for shaping conservation efforts in the face of evolving environmental challenges.

## 1. Introduction

Bats are vital contributors to biodiversity, playing an essential role in maintaining ecological balance and functioning through their diverse interactions and ecological services [1]. Insectivorous species contribute significantly to pest control. By feeding on pests, they help regulate pest populations and reduce crop damage, thereby minimizing the reliance on chemical pesticides for sustainable agriculture [2,3,4]. The abundance of bats in these agricultural lands is fundamental for the effective provision of pest control services, and any factor that influences the number of bats present at agricultural lands or the timing of foraging in these areas will have the potential to disrupt bat–insect interactions, thereby impacting their ability to provide essential pest control services [5].

A variety of stressors affect bats, with environmental conditions playing a significant role [6,7,8,9,10]. Factors such as wind speed, temperature fluctuations, rainfall, and relative humidity may impact the activity patterns of bats in foraging sites [7,11,12,13,14,15]. For example, high air temperatures are linked to increased insect availability [16,17] and have been associated with higher levels of activity by insectivorous bats [17,18,19,20]. Moreover, the association extends to the emergence patterns (the time when bats leave their roosts to forage during the night), with bats emerging later in warmer evenings compared to colder evenings, a phenomenon likely driven by the increased foraging success on hot days because of the underlying relationship with nocturnal insect activity and temperature [7]. This would allow bats to emerge later in the evening while still fulfilling their energy requirements, a pattern supported by several studies [7,14,15,17]. High wind speeds and heavy rain can potentially delay bat emergence and reduce flight patterns due to constraints imposed by raindrops on echolocation and the increased energetic demands of flight [21]. These weather conditions add additional costs for thermoregulation, particularly when there is decreased food availability [13]. Moreover, changes in the relative humidity of the air (RH) can influence insect activity [22], subsequently affecting bat flight activity. Relative humidity can impact bat echolocation performance, potentially hindering foraging activity and flight ability [23,24,25]. Specifically, high humidity levels can reduce the range and clarity of echolocation calls, posing challenges for high-frequency echolocator species in detecting and capturing prey. Consequently, in such conditions, species may exhibit reduced flight activity or alter their foraging strategies compared to low-frequency echolocator bats, to compensate for the limitations imposed by high humidity [24]. However, the effects of humidity are generally weaker than temperature and are species-specific [13,26,27,28,29].

The central region of Chile boasts a temperate Mediterranean climate, hosting a bat fauna predominantly composed of insectivorous species [30]. Despite the documented presence of various bat species in agricultural lands [31,32,33] and their recognized role as suppressors of pest insects [3], there are significant gaps in our understanding of how environmental factors impact bat activity at their foraging sites. Bridging these knowledge gaps is crucial for the strategic planning and design of research and monitoring programs aimed at preserving their populations and the associated ecosystem services, particularly in the face of climate change adaptation [34,35,36]. Chile, particularly in its Mediterranean regions, is expected to undergo diverse climatic changes in the coming decades due to global climate change [34,36,37]. Some of the potential impacts include an overall increase in temperatures, which could directly affect local ecology by altering seasonal patterns and environmental conditions for both plants and animals [38]. Projections also suggest variability in precipitation patterns, with the possibility of more intense droughts or extreme rainfall events. In this context, understanding how environmental factors influence bat activity is crucial for anticipating potential impacts on their populations and for promoting effective conservation strategies in the future. The present study aims to investigate the activity patterns of five species of insectivorous bats in a Mediterranean landscape dominated by vineyards in central Chile. We assess the influence of weather conditions, specifically air temperature and relative humidity, on the timing of activity and the relative bat abundance. We hypothesize that weather conditions, at dusk and during the night, will influence the timing of activity and relative abundance in foraging sites. Based on previous research, we predict that on warmer evenings, bats will initiate their activity at foraging sites later than on colder evenings. This is because foraging success is expected to be higher with increased temperatures, which in turn allows bats to emerge later in the evening while still fulfilling their energy requirements. Correspondingly, we predict bats will finish their activity earlier on warmer nights because they will be satiated in less time, as opposed to colder nights. Furthermore, we anticipate an increased bat relative abundance (expressed as relative activity) on warmer nights because of the underlying relationship with nocturnal insect activity and temperature. Finally, we also predict that responses of bats to relative humidity would be species-specific.

## 2. Materials and Methods

### 2.1. Study Area

The study was carried out in the locality of Huelquen, commune of Paine, Santiago Metropolitan Region, central Chile (33°48.412′ S, 70°39.086′ W to 33°51.960′ S, 70°35.352′ W). This region is characterized by typical Mediterranean agricultural landscapes, composed of a variety of crops, including horticultural crops, cereals, alfalfa fields, and orchards, and large extensions of vineyard farms in addition to small patches of native vegetation (sclerophyllous forest or shrubland), abandoned plantings such as *Eucalyptus* spp. and pine plantations (*Pinus* sp.), and urbanized or semiurban areas [31]. The climate of the region is temperate pluviseasonal Mediterranean according to the Köppen climate classification, with most rainfall concentrated in the winter season, June–August. The mean annual precipitation is ca. 360 mm and mean annual temperature is ca. 15 °C [39].

### 2.2. Bat Sampling

Automatic detectors (Song Meter SM4 BAT FS, Wildlife Acoustics, Maynard, MA, USA) coupled to external omnidirectional SMM-U1 ultrasonic microphones (Wildlife Acoustics, Inc., Maynard, MA, USA) were used to record bat echolocation calls. The detectors were positioned at seven sampling points within vineyard farms, ensuring a minimum separation distance of 1.0 km between each point. The recording parameters were set at a 256 kHz sampling rate, 16 kHz digital high-pass filter, and 18 dB trigger level. A 3.0 s trigger window was configured to capture calls preceding the initial trigger. The detectors operated throughout the night, starting 30 min before sunset (between 20:00 and 20:50 h) and ending 30 min after sunrise (between 06:00 and 06:59 h).

At each sampling point, bat activity was monitored for a minimum of 17 and a maximum of 25 nights, for a total of 143 sampling nights. The acoustic data collection spanned from October 2017 to February 2018, coinciding with spring and summer months in the southern hemisphere. This period aligns with the peak of bat activity seasons in Chile, as documented in [40].

Bat calls were displayed using BatSound 2.1 (Pettersson Elektronik AB, Uppsala, Sweden) and classified according to species by comparing the parameters of our recorded calls (duration, final frequency, slope frequency modulation, peak frequency, minimal and maximal frequency) to a library of validated reference calls from Chilean bats [30,41]. For each sampling night, the onset and end time of bat activity was recorded in minutes after sunset and minutes before sunrise, respectively. The onset time was identified as the moment of the first detected bat pass at a sampling point, and the end time was identified as the moment of the last detected bat pass. Bat relative abundance was evaluated using the Acoustic Activity Index (AI) proposed by [42], calculated as the sum of one-minute time blocks during which bat passes were detected at each sampling point. A bat pass was defined as a recording of 15 s maximum in which two or more pulses emitted by a bat were identified. Time of sunset and time of sunrise for Santiago Metropolitan Region were obtained from the meteorology portal Meteored (https://www.meteored.cl/, accessed on 21 December 2020).

### 2.3. Weather Conditions

Hourly average air temperature (°C) and relative humidity (%) between October 2017 and February 2018 were obtained from the Climatological Station in Huelquen, Paine (33°51′43.00″ S, 70°38′21.00″ W), administered by the Chilean Meteorological Directorate. Climate variables were assumed to not differ between the sampling points and the climatological station, because of the great homogeneity in the weather conditions of the area and the geographical proximity between them (maximum distance of 6.0 km). Weather variables were categorized into two periods: two hours before sunset (time immediately before bat activity) and 10 h after sunset (time of bat activity). Other factors such as rainfalls are very infrequent in central Chile during spring and summer [39]. Consequently, precipitation was not considered a reliable predictor and was excluded as an explanatory variable in our study. Similarly, wind speeds did not exhibit significant variations either between nights or within the same night throughout our study period, as indicated by the data from the climatological station; hence, it was not included as a predictor.

### 2.4. Statistical Analyses

The influence of weather conditions (air temperature and relative humidity) on the timing of activity and bat relative abundance was evaluated using species-specific generalized additive mixed models (GAMMs), with a negative binomial and log-link function. GAMMs extend the concept of linear regression by including smoothing functions to fit non-linear relationships between a response variable and predictor variables [43]. Analyses were conducted using the *gam* function in the *mgcv* package of the statistical software RStudio (version 2022.02.1; RStudio, Inc., Boston, MA, USA, www.rstudio.com, accessed on 17 July 2023). Weather variables were included in all models as fixed factors. Air temperature and relative humidity values before sunset were used as predictor when modeling the onset time of bat activity, while overnight air temperature and relative humidity were used for modeling the end time and bat relative abundance. The onset time was also included as a fixed factor in the species models to assess a potential effect of this variable on the ending of the activity. Each sampling point was included in all models as a random factor (site, bs = “re”). All predictor variables were standardized to a mean of zero and a standard deviation of one to give all variables equal weight and to simplify expected variance partitions for each predictor [44].

For each response variable, models were compared with every possible combination of predictor variables and ranked in relation to each other using the Akaike information criteria [45], delta (e.g., differences between best and secondary models with significant results). Akaike weights were calculated to assess the likelihood of the model relative to the other models considered. From the models where the delta value was <2, the principle of parsimony was used to find the model that best explained the variation in the data with the lowest number of predictor variables included [46]. For simplicity, here, we present the results of the most parsimonious models (lowest AIC). Possible alternative models within a 95% confidence interval based on AIC weights (relative probabilities of each model being the best model) are shown in Supplementary Materials. Model fit was interpreted as the proportion of the total deviance explained (D^2^) using script adapted from Elith et al. [47].

## 3. Results

A total of 10,038 echolocation passes were recorded, with 99.6% (10,001 passes) being identifiable and assignable to one of the six species monitored in the study area: Brazilian Free-tailed Bat *Tadarida brasiliensis* (7768 passes), Southern Hoary Bat *Lasiurus villosissimus* (719 passes), Valparaiso Myotis *Myotis arescens* (676 passes), Cinnamon Red Bat *Lasiurus varius* (561 passes), Small Big-eared Brown Bat *Histiotus montanus* (254 passes), and Big-eared Brown Bat *Histiotus macrotus* (23 passes). We recorded less than 100 bat passes of *H. macrotus* and therefore excluded this species from the analysis.

**Figure 1:**
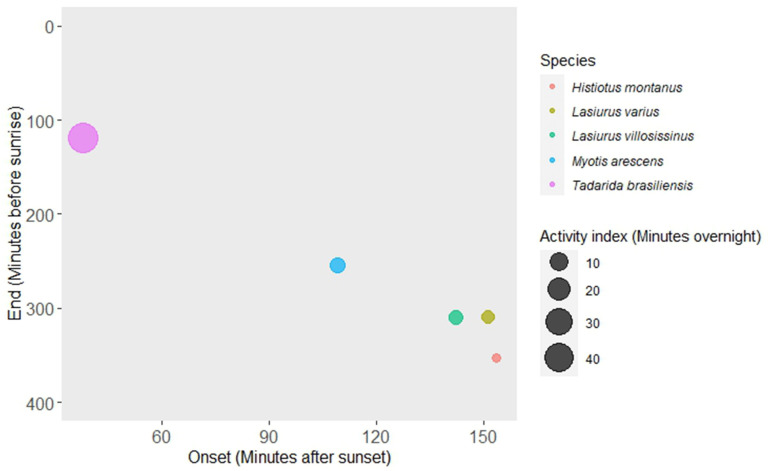
Relationships between the average onset (*x*-axis) and end time (*y*-axis) of bat activity and the relative abundance (circle size) in the agricultural landscape in central Chile.

The activity of *T. brasiliensis* was notable for its early onset, with the first bat pass detected occurring, on average, 37.80 min ± 1.31 SE after sunset. Additionally, this species exhibited a delayed withdrawal from foraging sites (118.44 min ± 8.21 SE before sunrise), following all other species (Figure 1 and Table 1). *Tadarida brasiliensis* also displayed a higher relative abundance compared to the other four species, with an average of 44.77 min ± 3.38 SE of bat passes recorded per night (Figure 1 and Table 1). An early onset in foraging activity was significantly associated with an early end of the activity for most species (*M. arescens*, *L. varius*, *L. villosissimus*, and *H. montanus*) in the foraging sites (Table 2), suggesting a limited time window for foraging.

**Figure 2:**
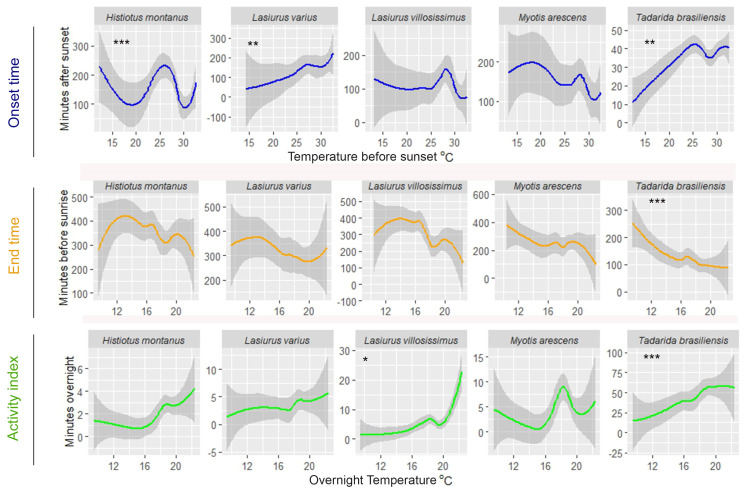
Observed changes in the onset and end time of bat activity, and the relative abundance per species in relation to air temperature in the agricultural landscape in central Chile. Gray-shaded areas indicate 95% confidence intervals. Asterisks indicate a significant relationship between the predictor and the response variable. Significance codes: *** 0.001, ** 0.01, * 0.05.

**Figure 3:**
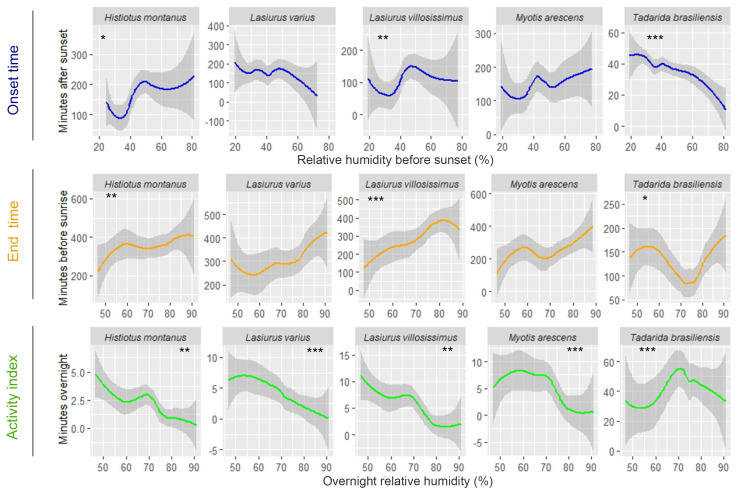
Observed changes in the onset and end time of bat activity, and the relative abundance per species in relation to relative humidity in vineyards in the agricultural landscape in central Chile. Gray-shaded areas indicate 95% confidence intervals. Asterisks indicate a significant relationship between the predictor and the response variable. Significance codes: *** 0.001, ** 0.01, * 0.05.

Examining the impact of weather conditions on the timing of bat activity, *Lasiurus varius* and *T. brasiliensis* showed a positive linear relationship with air temperature before sunset, initiating their activity earlier on colder evenings compared to warmer evenings (Figure 2 and Table 3). *Histiotus montanus* exhibits a zigzag-shaped response to temperature changes before sunset. On colder evenings with temperatures below 15 °C and on moderately warm evenings, around 25 °C, this bat tended to initiate its activity later. Also, this species delays the onset of activity on the hottest evenings, with temperatures exceeding 30 °C (Figure 2). Among these species, only *T. brasiliensis* responded to variations in overnight air temperature, remaining active until later (closer to dawn) on warmer nights compared to colder nights (Figure 2 and Table 2). A significant relationship with relative humidity before sunset was also observed in *T. brasiliensis*, as it initiates earlier on more humid evenings compared to drier ones (Figure 3 and Table 3). This species also remained active until later, on nights with a peak of overnight relative humidity values between 70 and 80%, compared to more humid or drier nights (Figure 3). An early onset occurred on drier evenings compared to more humid evenings in *H. montanus* (Figure 3). Similarly, *L. villosissimus* initiated its activity earlier on evenings with relative humidity below 40% and above 60%, delaying its onset as much as possible on evenings with RH close to 50% (Figure 3). Both *H. montanus* and *L. villosissimus* finished their activity significantly later, on drier nights than on more humid nights (Figure 3).

The relative abundance of most bat species was also significantly influenced by overnight temperature and relative humidity. Specifically, the number of minutes with bat passes of *T. brasiliensis* and *L. villosissimus* increased on warmer nights compared to colder nights (Figure 2 and Table 4). Additionally, *L. varius*, *L. villosissimus*, *H. montanus*, and *M. arescens* exhibited increased activity on drier nights compared to more humid nights (Figure 3 and Table 4). Notably, *T. brasiliensis* displayed a peak on nights with relative humidity values close to 70%, decreasing its activity at higher or lower relative humidity values (Figure 3 and Table 4).

## 4. Discussion

Our results reveal several patterns related to the activity of five species of insectivorous bats in a Mediterranean landscape in central Chile and how this activity is influenced by weather conditions, specifically air temperature and relative humidity. We found that *Tadarida brasiliensis* stood out for its early activity compared to the other four species (*M. arescens*, *H. montaus*, *L. villosssimus*, and *L. varius*). In North America, the emergence timing of *T. brasiliensis* exhibits significant variation from year to year, influenced by climate and weather conditions, as demonstrated by Frick et al. [7]. Based on data collected from five maternity colonies in Texas (United States), these researchers observed that during summers marked by severe drought conditions, the bats tended to emerge as early as 100 min before sunset. In contrast, in moist summers, they emerged as late as 43 min after sunset. The average onset time of *T. brasiliensis* in our study (37.80 min ± 1.31 SE after sunset) fall within these and other ranges previously described [15,48], highlighting the consistency of our findings with the existing data. The remaining four species were detected, on average, 96 min after sunset and represent the first known activity onset values for these species. The timing of bats’ emergence is closely linked to minimizing the risk of predation and is a key aspect of their behavior and ecology that allows them to survive and thrive in their environment [29,49,50]. This level of predation risk differs for species that hunt in open, cleared habitats, compared to those that forage along forest edges or in cluttered environments, potentially resulting in different responses to the prevailing twilight light levels at the time of the emergence [51]. Fast-flying aerial-hawking species, typically found in open habitats, are less constrained by light-dependent predation risk and tend to tolerate twilight light levels and, consequently, tend to emerge earlier [51]. A prime example of this is *T. brasiliensis*, which, like other open-space foragers, showcases swift flight capabilities. This reduced vulnerability to predation may explain the early emergence of this species when compared to edge-space or narrow-space foragers such as *L. varius*, *L. villosissimus, M. arescens*, and *H. montanus* in our study [52,53].

Notably, *T. brasiliensis* also exhibited a late end of its activity and a higher relative abundance compared to the other species. The early onset and the late end of activity in this species may suggest temporal niche segregation from other bat species in our study area, allowing them to avoid competition and exploit resources more effectively [54,55]. To gain a deeper insight into the ecological dynamics at play, it is recommended to conduct more detailed studies on the ecology and behavior of these bat species to better understand the reasons behind temporal niche segregation. In contrast to the relatively high occurrences of *T. brasiliensis*, echolocation passes of *H. montanus*, *L. villosissimus*, *L. varius*, and *M. arescens* accounted for 22% of the total recorded. *Tadarida brasiliensis* is a commonly observed species in Chile, frequently found in both urban and rural environments, and is often documented in association with human habitats [32,33,56,57]. Our study sites are located on the outskirts of a metropolis (Santiago de Chile), raising the possibility that urbanization contributes to the observed high activity of this species compared to the others. *Histiotus montanus*, *M. arescens*, *L. villosissimus*, and *L. varius* are typically associated with forested habitats. *Lasiurus varius* and *L. villosissmus* exclusively roost in the foliage of trees, while *M. arescens* and *H. montanus* may occasionally use forests for roosting [30]. Consequently, it is plausible that a reduced availability of roosting sites may be constraining their abundance in our study sites, which has experienced significant loss of forest cover. Another explanation for the disproportionate activity of *T. brasiliensis* could be attributed to variations in the detectability of calls among species. Factors such as frequency range, intensity, and duration of the calls play a role in this detectability variation [58,59]. If a particular bat species has echolocation calls that are more easily detected by microphones, it could lead to an overestimation of its abundance compared to species with less detectable calls [59]. Thus, variations in activity values could arise from differences in the probability of detection rather than from actual variations in activity levels [60,61]. Unfortunately, we lack specific data on the intensity of calls for the bat species in our study sites. Without information on call intensity, it becomes challenging to unequivocally associate higher recorded activity with better detectability. Nevertheless, recognizing this limitation underscores the importance of conducting comprehensive studies that not only focus on abundance, but also delve into the specific acoustic traits of each bat species. Future research efforts should aim to gather detailed information on call characteristics, enabling a more nuanced understanding of bat population dynamics by disentangling actual activity levels from detection biases.

There was a clear influence of weather conditions on the timing and intensity of bat activity at foraging sites. We predicted that bats would initiate their activity later during warmer sunsets, based on the assumption that an increase in temperature would result in higher foraging success, as prey tends to be more abundant in warmer conditions. Consequently, bats could delay the onset of their foraging activities into the evening and still satisfy their energy requirements [7]. We found that, when significant, the effects of temperature at sunset were mixed across species; *L. varius* and *T. brasiliensis* delayed their onset during the warmest evenings, while *H. montanus* exhibited a delayed onset of activity during extremely cold and hot nights yet an earlier onset of activity on evenings characterized by moderate temperatures. These results support our predictions for *L. varius* and *T. brasiliensis*, but not for *H. montanus*. This finding could hold ecological implications for *H. montanus*, hinting at its ability to adapt its behavior to optimize foraging success. Such behavioral flexibility becomes increasingly relevant in the context of climate change, shedding light on how bat populations may adapt to and cope with long-term temperature fluctuations.

Overnight temperature also influenced the time when *T. brasiliensis* finishes its activity and the number of minutes with bat passes detected at foraging sites, while it had no discernible impact on the remaining four species. We predicted that bats would finish their activity earlier on warmer nights. This prediction was based on the assumption that foraging success is higher in hot days and bats will be satiated in less time, as opposed to colder nights. Instead, we found that during colder nights, *T. brasiliensis* finished its activity earlier compared to warmer nights. Maintaining bat flight requires continuous energy expenditure, increasing their energy consumption [62]. To cope with this, some bat species are not active all night, dividing their activity into feeding periods separated by intervals of nocturnal rest [11,63,64,65]. The time spent roosting at night and foraging varies depending on environmental conditions, where long night rest periods and short feeding periods are associated with cool nights and low prey density [11,14,15]. The cold temperatures recorded on some spring/summers nights in our study likely reduced insect availability, resulting in limited foraging successful on these nights. Cold ambient temperature would also have increased the energy cost associated with thermoregulation [62]. Therefore, low temperatures probably caused bats to shorten their foraging periods, thereby minimizing energy losses during periods of food scarcity. The return to more typical spring/summer environmental conditions probably resulted in prolonged activity over time to compensate for the loss in foraging activity. This result confirms suggestions from previous studies that *T. brasiliensis* can adapt its emergence behavior and feeding activity depending on the prevailing climatic conditions [7,15,48].

The impact of relative humidity on the timing of bat activity was another interesting finding in our study. As occurring with temperature, we found that when there was a significant effect of relative humidity at sunset, the results were mixed across species: *H. montanus* and *L. villossismus* were detected earlier on drier evenings compared to more humid evenings, while the opposite was observed for *T. brasiliensis*. Frick et al. [7] reported early emergence (sometimes well before sunset) in Brazilian free-tailed bats during severe drought conditions compared to moist conditions, referring to dry or moist periods within the same season (summer) in different years. This behavior has been interpreted as a potential compensatory mechanism, where bats respond to adverse conditions by extending their foraging time without suffering loss of fitness [7]. Drought refers to a prolonged shortage of rainfall that negatively affects the land, while low relative humidity refers to the percentage of water vapor in the air compared to the maximum amount the air could hold at a specific temperature. Drought directly impacts the availability of water in the environment, which can critically affect bats that rely on water sources for drinking and for their habitat and has also been linked to lower reproductive success and lower annual survival [66]. Low humidity can also influence water availability in the air and vegetation, which can also affect bats, although less directly [26,67]. Our study was confined to a single calendar year, limiting our ability to draw comparisons between dry and moist years. During this period, relative humidity values at our study site ranged from 30 to 80%, encompassing a spectrum from moderately low to high levels, which would likely not be associated with stressful conditions for Brazilian free-tailed bats. Furthermore, the negative correlation between temperature and relative humidity values at dusk and during the night (Pearson’s r = −0.75 and r = −0.67; *p* ≤ 0.001) indicates that the driest evenings and nights were also the hottest. Hence, the higher temperatures on these drier nights likely led to a delay in the onset of activity for Brazilian free-tailed bats, attributed to the increased foraging success expected on warmer nights, as mentioned before.

Relative humidity also significantly influenced the bat relative abundance in our study, with species such as *L. varius*, *L. villosissimus*, *M. arescens*, and *H. montanus* exhibiting decreased activity on more humid nights compared to drier ones. Atmospheric attenuation, encompassing the absorption, scattering, and reduction in sound intensity as it travels through the air, is a phenomenon particularly pronounced in high humidity conditions, where water vapor effectively absorbs specific wavelengths of light and sound [68]. High-frequency sounds are more susceptible to atmospheric attenuation than low-frequency sounds, affecting bats’ prey detection [69,70,71,72]. Notably, in our study, all species, except *T. brasiliensis*, which emits calls at a peak frequency of 24 kHz, are high-frequency echolocators (peak frequency values ≥ 30 kHz) [30,41]. This distinction becomes crucial when considering the possibility that on drier nights, atmospheric conditions may favor the detection and hunting of prey in these high-frequency echolocator species, potentially resulting in increased activity compared to nights characterized by higher atmospheric humidity.

High humidity levels are known to significantly influence insect behavior [22], and may therefore indirectly affect bats. While there are existing data on the nocturnal insect community at our study sites [73], it is limited and typically spans only one to three sampling nights. This constraint hindered the ability to understand how insect activities are controlled by humidity and temperature. The scarcity of long-term observations under-scores the need for more extensive and continuous monitoring to unravel the dynamics between weather conditions and the activities of nocturnal insects and bats. As we strive to understand these complexities, a key question emerges: are bats directly driven by temperature or indirectly influenced by prey availability? Addressing this question in future research will help in the development of tailored management strategies to meet the specific needs of bats in changing environments. Additionally, in the context of climate change, understanding whether temperature and humidity affects food resources of bats provides crucial insights for anticipating and mitigating the impacts of global warming on their populations and behaviors.

## 5. Conclusions

In summary, our study emphasizes the importance of understanding the interactions between insectivorous bat species and environmental factors in agricultural landscapes. Weather conditions, specifically temperature and relative humidity, played a significant role in shaping bat activity. Temperature influenced the onset of bat activity, supporting predictions for some species but challenging expectations for others, particularly *H. montanus*, showcasing behavioral flexibility. This adaptability is crucial in the context of climate change, providing insights into how bat populations may respond to temperature fluctuations over time. The impact of overnight temperature on the activity of *T. brasiliensis* revealed a link between climatic conditions and foraging behavior, emphasizing the species’ ability to adjust activity times to optimize energy expenditure. The impact of relative humidity on bat activity was notable, affecting both the timing of activity and relative abundance for various species. In light of these findings, we recommend further detailed studies on the ecology and behavior of these bat species, including investigations into prey preferences, habitat selection, and foraging strategies. Additionally, exploring the long-term impact of climate change on bat populations and their adaptive strategies can provide valuable insights for conservation efforts.

## Figures and Tables

**Table 1:** Summary statistics (mean ± SE) for the onset and end time of bat activity and the relative abundance by species in the agricultural landscape in central Chile. The numbers in parentheses represent the maximum and minimum values.

	Onset Time (Minutes after Sunset)	End Time (Minutes before Sunrise)	Activity Index (Minutes Overnight)
*Tadarida brasiliensis*	37.0 ± 1.31 (113–0)	118.44 ± 8.21 (541–22)	44.77 ± 3.38 (271–1)
*Myotis arescens*	142.29 ± 9.56 (529–17)	254.43 ± 13.45 (618–50)	4.41 ± 0.76 (54–0)
*Lasiurus varius*	151.07 ± 11.84 (514–24)	309.84 ± 15.77 (600–55)	3.50 ± 0.54 (41–0)
*Lasiurus villosissimus*	109.00 ± 9.63 (592–27)	309.06 ± 15.00 (620–23)	4.62 ± 0.55 (32–0)
*Histiotus montanus*	153.49 ± 9.50 (528–41)	353.21 ± 12.14 (595–91)	1.89 ± 0.24 (19–0)

**Table 2:** Summary table of the effect of predictors on the end time of bat activity (minutes before sunrise) in the most parsimonious GAMMs (lowest AIC). Possible alternative models (95% confidence set based on model wAIC) are shown in Supplementary Materials. Effective degrees of freedom (edf = 1.00 represents a linear relationship), percentage variance explained, and significance level are provided for each model. TeNo = Overnight temperature; HuNo = overnight relative humidity; Onset = onset time of activity of the species.

	Deviance Explained (%)	Predictor Variable	edf	χ^2^	*p*
*Tadarida brasiliensis*	26.9	Onset	2.36	6.91	0.100
		HuNo	1.92	16.75	≤0.05
		TeNo	1.00	25.94	≤0.001
*Myotis arescens*	16.3	Onset	3.01	9.52	≤0.05
		HuNo	1.00	3.80	0.051
*Lasiurus varius*	18.3	Onset	1.00	14.21	≤0.001
		HuNo	1.40	1.92	0.223
*Lasiurus villosissimus*	21.9	Onset	2.73	21.13	≤0.001
		HuNo	1.00	18.18	≤0.001
*Histiotus montanus*	31.0	Onset	1.57	41.49	≤0.001
		HuNo	1.00	10.63	≤0.01

**Table 3:** Summary table of the effect of predictors on the onset time of bat activity (minutes after sunset) in the most parsimonious GAMMs (lowest AIC). Possible alternative models (95% confidence set based on model wAIC) are shown in Supplementary Materials. Effective degrees of freedom (edf = 1.00 represents a linear relationship), percentage variance explained, and significance level are provided for each model. TeSu = Temperature before sunset; HuSu = relative humidity before sunset.

	Deviance Explained (%)	Predictor Variable	edf	χ^2^	*p*
*Tadarida brasiliensis*	31.9	HuSu	1.00	52.55	≤0.001
		TeSu	1.86	39.91	≤0.001
*Myotis arescens*	3.35	TeSu	1.00	3.20	0.074
*Lasiurus varius*	8.89	HuSu	1.00	2.55	0.110
		TeSu	1.00	10.28	≤0.01
*Lasiurus villosissimus*	11.1	HuSu	2.99	10.35	≤0.01
*Histiotus montanus*	35.2	HuSu	1.82	7.52	≤0.05
		TeSu	5.46	23.87	≤0.001

**Table 4:** Summary table of the effect of predictors on the bat relative abundance (number of one-minute time blocks during which bat passes were detected) in the most parsimonious GAMMs (lowest AIC). Possible alternative models (95% confidence set based on model wAIC) are shown in Supplementary Materials. Effective degrees of freedom (edf = 1.00 represents a linear relationship), percentage variance explained, and significance level are provided for each model. TeNo = Overnight temperature; HuNo = overnight relative humidity.

	Deviance Explained (%)	Predictor Variable	edf	χ^2^	*p*
*Tadarida brasiliensis*	28.7	HuNo	1.00	40.86	≤0.001
		TeNo	1.00	67.88	≤0.001
*Myotis arescens*	29.3	HuNo	2.98	22.01	≤0.001
		TeNo	5.56	5.57	0.081
*Lasiurus varius*	16.4	HuNo	2.46	23.17	≤0.001
		TeNo	1.03	8.11	0.078
*Lasiurus villosissimus*	24.8	HuNo	3.49	19.66	≤0.001
		TeNo	4.94	12.44	≤0.05
*Histiotus montanus*	13.2	HuNo	2.01	20.29	≤0.001

## Data Availability

The data presented in this study are available on request from the corresponding author. The data are not publicly available due to planned research in the future.

## Supplementary Materials

The following supporting information can be downloaded at: https://www.mdpi.com/article/10.3390/ani14060860/s1, Table S1: Summary of GAMM model selection statistic for evaluating the influence of weather conditions on the onset and end time of bat activity and relative abundance in a Mediterranean agricultural landscape in central Chile. The first model listed for each species is the minimum AIC model. Only models with a Δi of 2 or less are included as competing models. df: Degrees of freedom; AIC: Akaike information criteria; Δi: differences in AIC with respect to the top-ranked model; Wi: Akaike weight (relative probability of each model being the best model). TeSu = Temperature before sunset; HuSu = relative humidity before sunset; TeNo = overnight temperature; HuNo = overnight relative humidity; Onset = onset time of activity of the species.
