# Supplementary material for: Species-Specific Responses of Insectivorous Bats to Weather Conditions in Central Chile
Source: Animals (Basel). 2024 Mar 11;14(6):860. doi: 10.3390/ani14060860 (PMC10967300; doi:10.3390/ani14060860)
Supplement: Supplementary file 1 [file animals-14-00860-s001.zip › animals-2889298-supplementary.pdf]

Appendix A. Supplementary Material

Supplementary Tables

Table S1. Summary of GAMM model selection statistic for evaluating the influence of weather conditions on the onset and end time of bat activity and relative abundance in a Mediterranean agricultural landscape in central Chile. The first model listed at each species is the minimum AIC model. Only models with a Δ_i of 2 or less are included as competing models. df: degrees of freedom; AIC: Akaike information criteria; Δ_i: differences in AIC with respect to the top-ranked model; W_i: Akaike weight Relative probability of each model being the best model). *TeSu* = Temperature before sunset; *HuSu* = Relative humidity before sunset; *TeNo* = Overnight temperature; *HuNo* = Overnight relative humidity; *Onset* = Onset time of activity of the species.

**Response variable**	**Species /best-fitted models**	**df**	**AIC**	Δ_i	W_i
Onset time	*Tadarida brasiliensis*				
	s(HuSu)*** + s(TeSu)***	5.28	1294.68	0.00	0.53
	s(HuSu)***	10.74	1294.95	0.27	0.47
	Myotis arescens				
	s(TeSu)	3.01	1008.56	0.00	0.45
	s(HuSu)	3.00	1008.93	0.37	0.38

Response variable	Species /best-fitted models	df	AIC	Δ_i	W_i
	s(HuSu) + s(TeSu)	4.00	1010.48	1.92	0.17
	Lasiurus varius				
	s(HuSu) + s(TeSu)**	4.00	1160.26	0.00	0.56
	s(TeSu) **	3.00	1160.78	0.52	0.43
	Lasiurus villosissimus				
	s(HuSu) *	5.77	1135.60	0.00	0.62
	s(HuSu) + s(TeSu)	6.73	1137.55	1.95	0.23
	Histiotus montanus				
	s(HuSu)* + s(TeSu)***	10.90	1011.97	0.00	0.73
End time	*Tadarida brasiliensis*				
	s(Onset) + s(HuNo)* + s(TeNo)***	8.25	1528.70	0.00	0.71
	s(HuNo)*** + s(TeNo)***	5.06	1530.49	1.79	0.29
	Myotis arescens				
	s(Onset)* + s(HuNo)	6.76	1092.99	0.00	0.43

**Response variable**	**Species /best-fitted models**	**df**	**AIC**	**Δ_i**	**W_i**
	s(Onset)* + s(HuNo) + s(TeNo)	7.61	1094.82	1.83	0.17
	s(Onset) * + s(TeNo)	6.42	1094.85	1.87	0.17
	Lasiurus varius				
	s(Onset)*** + s(HuNo)	4.70	1353.57	0.00	0.35
	s(Onset)***	3.51	1353.95	0.38	0.29
	s(Onset)*** + s(TeNo)	4.00	1354.45	0.87	0.23
	s(Onset)*** + s(HuNo) + s(TeNo)	5.88	1355.54	1.97	0.13
	Lasiurus villosissimus				
	s(Onset)*** + s(HuNo)***	6.43	1380.45	0.00	0.69
	s(Onset)*** + s(HuNo)** + s(TeNo)	7.42	1382.24	1.79	0.28
	Histiotus montanus				
	s(Onset)*** + s(HuNo)**	4.96	1116.85	0.00	0.47
	s(Onset) *** + s(HuNo) + s(TeNo)	5.65	1117.50	0.66	0.34
	s(Onset) ***+ s(TeNo)*	4.75	1118.71	1.87	0.18

**Response variable**	**Species /best-fitted models**	**df**	**AIC**	$\boldsymbol{\Delta_i}$	$\boldsymbol{W_i}$
Activity index	*Tadarida brasiliensis*				
	s(HuNo) *** + s(TeNo) ***	4.00	1311.76	0.00	1.00
	Myotis arescens				
	s(HuNo) *** + s(TeNo)	12.40	641.02	0.00	0.99
	Lasiurus varius				
	s(HuNo)*** + s(TeNo)	6.18	636.81	0.00	0.66
	s(HuNo)***	4.73	638.12	1.31	0.34
	Lasiurus villosissimus				
	s(HuNo)*** + s(TeNo)*	12.29	715.24	0.00	0.93
	Histiotus montanus				
	s(HuNo)***	4.25	516.06	0.00	0.64
	s(HuNo)* + s(TeNo)	7.98	517.35	0.52	0.33
